# An image deblurring method using improved U-Net model based on multilayer fusion and attention mechanism

**DOI:** 10.1038/s41598-023-47768-4

**Published:** 2023-12-04

**Authors:** Zuozheng Lian, Haizhen Wang

**Affiliations:** College of Computer and Control Engineering, Qiqihar University, Qiqihar, 161006 China

**Keywords:** Computer science, Information technology

## Abstract

The investigation of image deblurring techniques in dynamic scenes represents a prominent area of research. Recently, deep learning technology has gained extensive traction within the field of image deblurring methodologies. However, such methods often suffer from limited inherent interconnections across various hierarchical levels, resulting in inadequate receptive fields and suboptimal deblurring outcomes. In U-Net, a more adaptable approach is employed, integrating diverse levels of features effectively. Such design not only significantly reduces the number of parameters but also maintains an acceptable accuracy range. Based on such advantages, an improved U-Net model for enhancing the image deblurring effect was proposed in the present study. Firstly, the model structure was designed, incorporating two key components: the MLFF (multilayer feature fusion) module and the DMRFAB (dense multi-receptive field attention block). The aim of these modules is to improve the feature extraction ability. The MLFF module facilitates the integration of feature information across various layers, while the DMRFAB module, enriched with an attention mechanism, extracts crucial and intricate image details, thereby enhancing the overall information extraction process. Finally, in combination with fast Fourier transform, the FRLF (Frequency Reconstruction Loss Function) was proposed. The FRLF obtains the frequency value of the image by reducing the frequency difference. The present experiment results reveal that the proposed method exhibited higher-quality visual effects. Specifically, for the GoPro dataset, the PSNR (peak signal-to-noise ratio) reached 31.53, while the SSIM (structural similarity index) attained a value of 0.948. Additionally, for the Real Blur dataset, the PSNR achieved 31.32, accompanied by an SSIM score of 0.934.

## Introduction

With the increasing popularity of mobile devices and multimedia communication, images have evolved into the primary carriers of information. Owing to both the inherent constraints of imaging systems and the dynamic and unpredictable nature of the shooting environment in dynamic scenes, image blurring becomes an inevitable occurrence^1^. Thus, research attention has shifted towards image deblurring methods. Blurred images not only subjectively affect the visual experience^2–4^, but also affect subsequent visual tasks^5^. Accordingly, addressing image deblurring techniques for dynamic scenes emerges as a crucial problem to solve^6^.

Conventional image deblurring methods rely on either a known or assumed blur kernel and leverage prior information about the image. However, these methods encounter challenges when dealing with the removal of blur induced by complex factors^7^. The development of deep neural networks has paved the way for blind image deblurring methods that do not necessitate the estimation of blur kernels. Thus, such methods have gained widespread use. Among the various deep neural network approaches, U-Net stands out in that an improved version of FCN (Fully Convolutional Neural Networks) is incorporated. This enhancement provides U-Net with increased flexibility in integrating features from multiple hierarchical levels, making it a powerful tool in image deblurring. By doing so, the complexity of deep networks is effectively reduced, significantly curtailing the number of parameters while maintaining accuracy within an acceptable range. Additionally, there is a scarcity of research on image deblurring based on U-Net, which provided motivation to explore an improved U-Net for solving image deblurring in dynamic scenes. The main objectives were as follows: (1) Investigate and develop an improved U-Net architecture tailored for image deblurring purposes; and (2) Examine and identify key modules capable of extracting image details and crucial information across different layers, thereby enhancing image feature extraction capabilities and improving visual outcomes.

Recently, U-Net and its improved versions are gradually being applied in image deblurring related fields such as image enhancement, image restoration, and super-resolution. For example, Liu et al.^8^ proposed Retinex-UNet, which uses convolutional neural networks to learn and decompose images. The results were input into the enhancement network for end-to-end training. The described method can enhance images of any size and improve the overall visual effect. Raj et al.^9^ proposed a residual dense connection-based U-Net model for fundus image enhancement, which effectively captures both local and global information. The experiment demonstrated that the proposed model could effectively enhance the visual quality of fundus images. Chen et al.^10^ introduced a U-Net-like deep stacked autoencoder neural network model designed for the restoration of images distorted by atmospheric turbulence. The model fuses low-level and high-level information, greatly ensuring the integrity of information, and obtaining high-quality restored images. Chen et al.^11^ proposed a deep learning method called NCS-Unet. This method incorporates distinctive features from the non-subsampled contourlet transform (NSCT) and the Sobel filter to extract valuable information. Consequently, it enhances the performance of noise reduction and artifact removal in PCT images. To improve low-resolution fundus images, Fan et al.^12^ proposed a style-guided U-Net, which incorporates a series of style-guided U-shape blocks (SUB). SUB enlarges the receptive field and fully fuses the hierarchical features. The experimental results demonstrated that SGUNet was more robust and accurate than other methods. Mao et al.^13^ proposed a Residual Fast Fourier Transform with Convolution Block and used it as the foundation for constructing a deep network. This network is capable of capturing both long-term and short-term interactions in the data while integrating low- and high-frequency residual information. Their experimental results demonstrated improved deblurring performance using this approach. Wu et al.^14^ proposed a U-Net model containing dense blocks for dynamic scene deblurring. The model significantly reduces the inference time.

Among the described methods, some^8–12^ are only suitable for image deblurring in specific situations, while others^13,14^ cannot achieve cross layer flow of feature information, and there is room for improvement in their feature extraction capabilities. The integration of wavelet transforms with deep convolutional neural networks is beneficial for mitigating image blur. Meanwhile, to overcome the vanishing gradient problem, DSC (depth-wise separable convolution)^15^ and residual networks^16^ can address the described problems. Additionally, skip connections in U-Net can sometimes introduce redundant information. Attention mechanisms have demonstrated their effectiveness in extracting critical and relevant information from the data^17–19^, providing a solution to this issue. Drawing inspiration from the previously mentioned techniques, the present study introduces a novel synthetic image deblurring method. This method combines wavelet transforms, DSC (depth-wise separable convolution), residual networks, and attention mechanisms to enhance the capabilities of the U-Net architecture. The aim is to effectively address the challenges associated with image deblurring.

The present study offers the following contributions: (1) A 4-layer network based on U-Net was proposed, including one encoder and one decoder with four blocks; (2) A MLFF module was added, which integrates feature information in different layers of the U-Net network, changes the inherent information flow mode in the conventional U-Net network, and integrates feature information of different scales, so that the network can extract more feature information; (3) A DMRFAB module introducing both CAM (channel attention mechanism) and SAM (spatial attention mechanism) was incorporated to extract crucial information from deep features and improve the image deblurring effect accordingly; (4) FFT was introduced into the loss function and FRLF was proposed, which allows for the frequency value of the image to be obtained by reducing the frequency difference of the image, thereby improving the deblurring effect. The proposed model underwent both quantitative and qualitative analysis using the GoPro and Real Blur datasets. The results reveal that the image deblurring quality was significantly enhanced by the proposed model.

The rest of the paper is organized as follows. The proposed methodology is presented in detail in section “The proposed method”. The experiment results and discussion are given in section “Results” and section “Discussion”, respectively. The conclusion is provided in section “Conclusion”.

## The proposed method

The proposed method was based on an improved U-Net model, which is discussed in this section.

### The model structure

**Figure 1:**
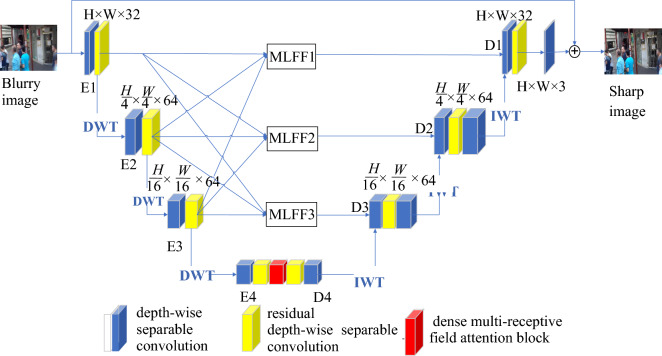
The improved model structure.

The proposed method was based on an improved U-Net model, of which the structure is shown in Fig. 1. The model includes one encoder and one decoder. The encoder uses four blocks to extract features from each layer, with a convolutional structure of 2-2-2-2. Every block, with the exception of the initial block, undergoes a specific process involving one down-sampling operation using Discrete Wavelet Transform (DWT) followed by two convolutional operations. The extracted features from each layer are fused through the MLFF module. The decoder adopts four blocks, with a convolutional structure of 2-3-3-2. Except for the last block, each block undergoes two convolutions and one Inverse Wavelet Transform (IWT). To reduce convolution parameters and computational complexity, and solve the degradation problem in deep networks, convolutional blocks are replaced with DSC (depth-wise separable convolutions) and RDSC (residual depth-wise separable convolutions). The encoder and decoder are connected through DMRFAB to allow for richer and more detailed features to be obtained.

The model is mainly composed of DSC, RDSC group including three RDSC, DWT, IWT, MLFF module, and DMRFAB module. The model is a 4-layer network based on U-Net, with the encoder on the left and the decoder on the right. The input image is represented as H × W × 3, where H represents the height of the image, W represents the width of the image, and 3 represents the number of channels in the image. The processing of the model involves the following three stages:

(1) In the encoding stage, from top to bottom, the first layer uses one 32-channel DSC and one RDSC group, which is composed of three RDSCs, and transforms the input image into H × W × 32, which is represented as E1. The second layer is entered through the first level DWT, using one 64-channel DSC and one RDSC group to transform the input feature information into $\frac{H}{4}\times \frac{w}{4}\times 64$, which is represented as E2. After entering the third layer through the second level DWT, one DSC and one RDSC group are used to transform the entered feature information into $\frac{H}{16}\times \frac{w}{16}\times 128$, which is represented as E3. After entering the fourth layer through the third level DWT, the incoming information is transformed into $\frac{H}{32}\times \frac{w}{32}\times 256$ using one DSC, one RDSC group, and one DMRFAM. Subsequently, the number of channels is reduced to 512 through DSC.

(2) The feature information of E1, E2, and E3 of the encoder is input into $MLFFi$ (i = 1,2,3) for fusion of different layers of feature information.

(3) In the decoding stage, IWT is performed on the output information D4 in the fourth layer, and passed through one DSC and one RDSC group with D3, where it is transformed into $\frac{H}{16}\times \frac{w}{16}\times 128$, followed by one more DSC operation to increase the number of channels to 256. The third layer outputs feature for IWT, and enters one DSC and one RDSC group with D2, transforming the fused information into $\frac{H}{4}\times \frac{w}{4}\times 64$. Subsequently, one DSC operation is performed to increase the number of channels to 128. The second layer outputs features for IWT, and D1 enters one DSC and one RDSC group, transforming the fused information into H × W × 32. The convolutional feature information that can be separated by depth becomes H × W × 3. This output is added and fused with the image information from the initial input model to obtain the deblurred image as the final result.

The modules of the model are introduced in detail below.

### DSC

**Figure 2:**
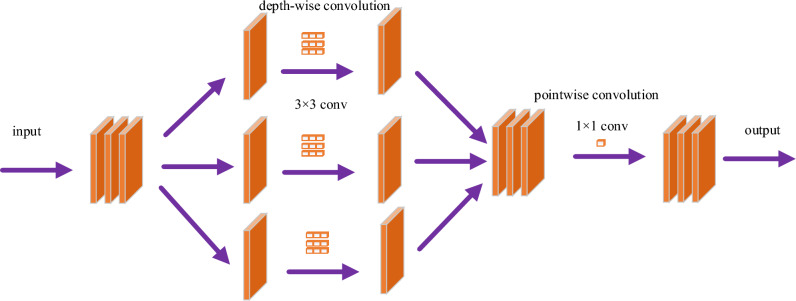
The structure of DSC.

The improved model introduces DSC, which decreases the number of the model parameters, and makes the network lightweight. The structure of the model is shown in Fig. 2. The DSC is composed of DWC (depth-wise convolution) and PWC (point-wise convolution). DWC divides the multi-channel features of the previous layer into the feature map of a single channel, and then uses a 3 × 3 convolution kernel for convolution. Subsequently, DWC recombines them, adjusting the size of the feature map from the previous layer while maintaining the same number of channels. The characteristic image attained by DWC is convoluted using PWC, which uses a 1 × 1 convolution kernel to blend the convolution results from DWC while having the flexibility to alter the number of output channels as needed.

### RDSC

**Figure 3:**
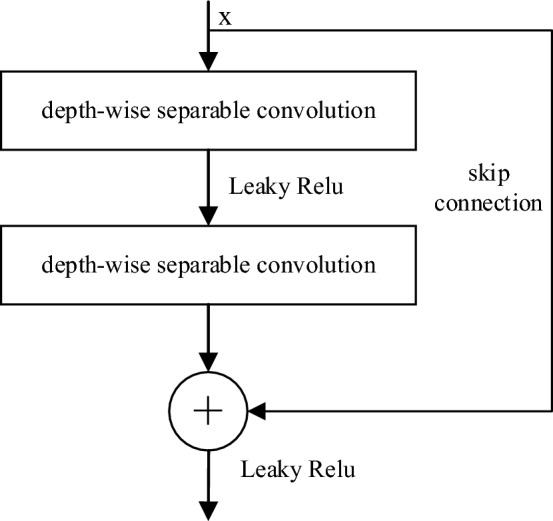
The structure of RDSC.

RDSC was designed based on the residual network, which can spread detailed information from different layers to promote blur reconstruction quality. It also serves as a mechanism to mitigate the issue of gradient vanishing. The RDSC uses two DSC and two Leaky Relu activation functions, and the structure is shown in Fig. 3. First, DSC, Leaky Relu, and DSC operations are performed for the input information. Then, the obtained features and input information are fused by means of skip connection. Finally, the fusion result is output after Leaky Relu processing.

### DWT and IWT

DWT and IWT respectively replace the down-sampling and up-sampling functions of the U-Net model, which can obtain image information at different frequencies, thereby reducing feature information loss during image reconstruction and further mitigating the blurring effect. As shown in Fig. 1, DWT and IWT are respectively performed on the results of E1, E2, and E3 in the encoder, and IWT is performed in the decoder. The Haar wavelet is a wavelet basis function which is easy to implement and operate. Therefore, in the present study, a two-dimensional Haar wavelet was adopted for wavelet transform operations to divide the image signal into directional sub-bands. Filtering is an effective method for realizing DWT. Firstly, a one-dimensional high-pass filter represented by $\varphi (x)$ (as defined in Eq. (1)) is utilized to filter and vertically down-sample each column of the image. Subsequently, both $\varphi (x)$ and $\psi (x)$ (defined in Eq. (2)) are employed to filter and horizontally down-sample each row. This process yields sub-frequency information for ${\text{I}}_{\text{HH}}$ and ${\text{I}}_{\text{HL}}$. In the second step, the $\psi (x)$ filter is used to filter and vertically down-sample each column of the image. Once again, $\varphi (x)$ and $\psi (x)$ are used to filter and horizontally down-sample each column. This results in sub-frequency information for ${\text{I}}_{\text{HH}}$ and ${\text{I}}_{\text{HL}}$. Sub-frequency information for the four parameters is shown in Eqs. (3)–(6).

1 $$\varphi \left(x\right)=[-\mathrm{1,1}]$$

2 $$\psi \left(x\right)=[\mathrm{1,1}]$$

3 $${\text{I}}_{\text{HL}}\left(x,y\right)=\varphi (x)\psi (y)$$

4 $${\text{I}}_{\text{HH}}\left(x,y\right)=\varphi (x)\varphi (y)$$

5 $${\text{I}}_{\text{LH}}\left(x,y\right)=\psi (x)\varphi (y)$$

6 $${\text{I}}_{\text{LL}}\left(x,y\right)=\psi (x)\psi (y)$$

The $x$ and $y$ in Eqs. (1)-(6) represent rows and columns for the information of the image; ${\text{I}}_{\text{HL}}$ denotes the horizontal high-frequency and vertical low-frequency information of the image; ${\text{I}}_{\text{HH}}$ denotes the horizontal and vertical high-frequency information of the image; ${\text{I}}_{\text{LL}}$ expresses the horizontal and vertical low-frequency information of the image; and ${\text{I}}_{\text{LH}}$ represents the horizontal low-frequency and vertical high-frequency image information. The IWT performs inverse operations on the four sub-images using the aforementioned filter. Thus, ${\text{I}}_{\text{HL}}$, ${\text{I}}_{\text{HH}}$, ${\text{I}}_{\text{LL}}$ and ${\text{I}}_{\text{LL}}$ are used to fuse into the original image. Therefore, the original image is decomposed by DWT and then reconstructed by IWT without loss of information. Further, multi-level wavelet transforms can be implemented by further processing ${\text{I}}_{\text{HL}}$, ${\text{I}}_{\text{HH}}$, ${\text{I}}_{\text{LL}}$ and ${\text{I}}_{\text{LL}}$ according to the described method. For the two-dimensional Haar wavelet transform, the sum mean value is used for low-frequency information, regarded as $\psi (x)$, while the difference in mean values is used for high-frequency information, regarded as $\varphi (x)$.

### MLFF module

**Figure 4:**
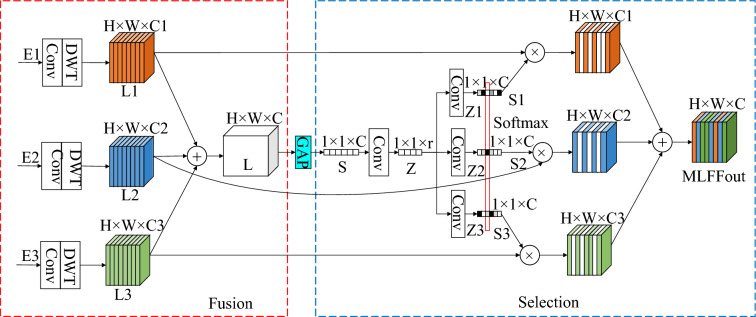
The structure of MLFF.

In the existing improved U-Net networks, the flow of feature information is inflexible, allowing for only horizontal information flow in the same layer, or vertical information flow between upper and lower layers. As such, the proposed model is different in that an MLFF module was designed, which increases the flow of information between different layers of U-Net, and integrates the characteristic information of different layers. However, a straightforward approach involving the addition or concatenation of these information sources can lead to redundancy in the fusion information and may restrict the expressive capacity of the neural network. Drawing inspiration from SKNets^20^, a dynamic selection mechanism was introduced to promote the expression ability of the network. Therefore, the MLFF module increases the flexibility of feature flow, which reduces the information redundancy of fusion, and improves the performance of the model. The method decreases the number of model parameters and produces a better effect than the simple cascade aggregation method. The structure of MLFF is shown in Fig. 4, including the cross-layer flow details of U-Net three-layer characteristic flow.

As shown in Fig. 4 L1 to L3 respectively represent Layer1 to Layer3 of the proposed model, and the MLFF module includes two stages: fusion and selection.

(1) Fusion stage: E1, E2 and E3 undergo convolution and wavelet transformation, respectively. The number of channels is controlled by convolution (Conv), and the feature size is controlled by wavelet transform (WT). This results in characteristic information represented as H × W × C for L1, L2, and L3. Subsequently, the feature elements of L1, L2, and L3 are added together to obtain the fused output L.

(2) Selection stage: S from L are obtained through global average pooling (GAP), so that the feature information is changed from H × W × C to 1 × 1 × C. The down channel convolution layer is used to change the feature vector S into a more compact feature vector Z, and the feature information changes from 1 × 1 × C to 1 × 1 × r (r = C/8). The feature vector Z is passed through the convolution layer of three parallel ascending channels to obtain three feature vectors Z1, Z2 and Z3, each of which has a feature size of 1 × 1 × C. The activation function Softmax is applied to Z1, Z2, and Z3 to obtain the activated S1, S2, and S3. Subsequently, S1, S2, S3 and L1, L2, and L3 point multiplication operations are respectively employed to adaptively calibrate L1, L2, and L3 feature maps. Finally, the calibrated features are fused to obtain the MLFF output. The output expression of MLFF is shown in Eq. (7).

7 $$MLFFout=S1\times L1+S2\times L2+S3\times L3$$

The model structure shows that there are three MLFF modules, namely $MLFF1$, $MLFF2$ and $MLFF3$. The modules differ only in the fusion part, that is, different layers have different feature transformations before feature fusion, while the subsequent selection parts are the same. MLFF1, MLFF2 and MLFF3 are represented as Eqs. (8)-(10).

8 $$MLFF1=E1\times {S1}^{1}+{(Conv\left(E2\right))}^{\downarrow }\times {S1}^{2}+{(Conv\left(E3\right))}^{\downarrow }\times {S1}^{3}$$

9 $$MLFF2={(Conv\left(E1\right))}^{\downarrow }\times {S2}^{1}+E2\times {S2}^{2}+{(Conv\left(E3\right))}^{\uparrow }\times {S2}^{3}$$

10 $$MLFF3={(Conv\left(E1\right))}^{\downarrow }\times {S3}^{1}+{(Conv\left(E2\right))}^{\downarrow }\times {S3}^{2}+E3\times {S3}^{3}$$

where $MLFFi$ represents the output of MLFF at layer i of the model; $Conv(\cdot )$ is the convolution kernel of 1 × 1, which is used to adjust the number of channels to facilitate the operation of wavelet transform; ↑ represents the feature information of the same level size obtained through the wavelet transform; ↓ represents the feature size of the same level obtained through the IWT; × and + respectively represent point multiplication and addition operations between feature elements; ${Si}^{j}$ represents the $MLFFi$ fusion multi-layer feature information obtained after the selection stage and activation, specifically the jth feature component; and the values of j and i are 1, 2, 3.

### DMRFAB module

**Figure 5:**
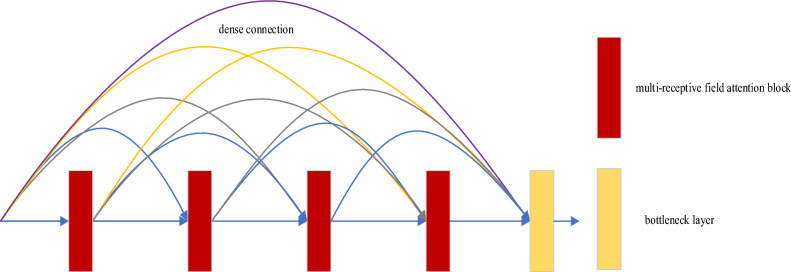
The DMRFAB module.

**Figure 6:**
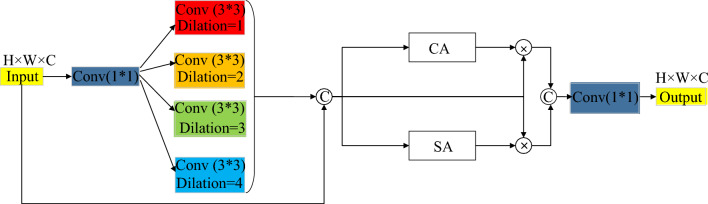
The structure of MRFAB.

In a CNN, the convolutional kernel processes the entire image uniformly without focusing on specific areas. Attention mechanisms can ignore certain irrelevant regional information and focus on the key areas in the image through learning. Different from other methods, the proposed DMRFAB module includes a dense multi-receptive field module both introducing SAM^21^ and CAM^22^, which helps multi- receptive field blocks better extract deep feature information, improve feature representation capabilities, and ultimately improve module deblurring performance. The DMRFAB module, illustrated in Fig. 5, comprises four MRFAB units and a bottleneck layer. The MRFAB units are responsible for extracting semantic features from the image, while the bottleneck layer reduces the number of feature inputs, enhancing the model's efficiency and compactness. A dense connection enhances the transmission of image features and makes more effective use of image features. The DMRFAB is shown in Eq. (11).

11 $${X}_{out}=G\left\{\left({H}_{i}\left[{x}_{0},{x}_{1},\dots ,{x}_{i-1}\right]\right);\upvarepsilon \right\}$$

where $\left[{x}_{0},{x}_{1},\dots ,{x}_{i-1}\right]$ indicates the feature map made by the DMRFAB of 0, 1, …, i − 1 layers in series; ${H}_{i}$ represents converting multiple input tensors into a single tensor; $G(\cdot )$ represents the output of the bottleneck layer; $\upvarepsilon$ is the super parameter of the bottleneck layer, and the filter size used in the bottleneck layer is 1 × 1. The structure of MRFAB used by the DMRFAB module is shown in Fig. 6.

As shown in Fig. 6, input information is firstly input into a convolution block using 1 × 1 convolution kernel, and then feature information with four feature extraction branches is extracted utilizing 3 × 3 convolution kernel with extensional rates of 1, 2, 3 and 4. The connection operation fuses the parallel feature maps of the five branches as shown in Eq. (12). The feature information fused through the connection operation is directed into two modules: the CA module, responsible for implementing the CAM, and the SA module, which implements the spatial attention mechanism. The outputs from these modules are individually point-multiplied with the fused information. Subsequently, the connection operation is applied once more. Finally, the input is processed by a convolution block using a 1 × 1 convolution kernel, as described in Eq. (13). This step serves to fuse and reduce the dimensionality of the feature information. In Eqs. (12), (13), R represents the feature map connecting different branch receptive fields; X represents the input of MRFAB; CA (•) represents the operation of the CA module; SA (•) represents the operation of the SA module; C $\mathrm{at}(\cdot )$ represents the connection operation; and M represents the output of MRFAB.

12 $$R=Cat\left[\begin{array}{c}\begin{array}{c}X*{W}_{1\times 1}*{W}_{3\times 3}^{d=1}\\ X*{W}_{1\times 1}*{W}_{3\times 3}^{d=2}\\ X*{W}_{1\times 1}*{W}_{3\times 3}^{d=3}\end{array}\\ X*{W}_{1\times 1}*{W}_{3\times 3}^{d=4}\\ X\end{array}\right]$$

13 $$M=Cat\left[CA\left(R\right)*R,SA\left(R\right)*R\right]*{W}_{1\times 1}$$

**Figure 7:**
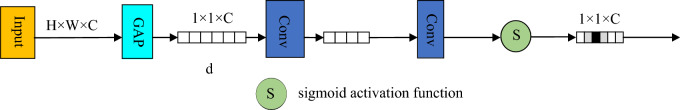
The Structure of CA module.

The CA module maps the relationship between feature channels through compression and excitation operations. The structure of the CA module, as depicted in Fig. 7, takes an input feature map G with dimensions H × W × C. First, it employs global average pooling (GAP) to compress the dimensions, resulting in a feature vector d with dimensions 1 × 1 × C, which encodes global context information. Then, the incoming features go through two convolutional layers followed by a sigmoid activation function. The CA module ultimately produces features with a size of 1 × 1 × C.

**Figure 8:**
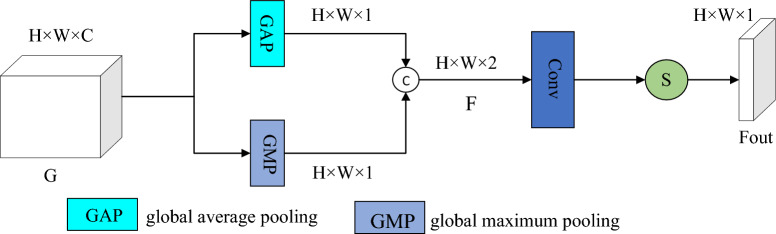
The Structure of SA module.

The SA module mainly uses the spatial correlation between features, and its structure is shown in Fig. 8.

The SA module can transform the input multi-dimensional features into a one-dimensional feature map with spatial characteristics, and correct the incoming feature information. The SA module takes as input a feature map G with dimensions H × W × C. It utilizes both maximum pooling and global average pooling, and then combines the results to form a feature map F with dimensions H × W × 2. Following this, a convolutional layer and sigmoid activation function are applied to generate the output feature map Fout with dimensions H × W × 1. The mathematical expression of the SA module is represented in Eq. (14).

14 $$Fout=Sigmoid(Conv\left(Cat\left[MAXPool\left(G\right),AvgPool\left(G\right)\right]\right))$$

where G indicates the input characteristics; MAXPool (•) indicates the global maximum pooling operation; AvgPool (•) indicates the global average pooling operation; Cat indicates the connection operation; Conv (•) indicates the convolution operation; and Sigmoid (•) indicates the activation function.

## Results

### Dataset and training details

In the present study, the GoPro and Real Blur datasets^23^ were used for the experiments. The GoPro dataset is composed of 3214 clear and blurred image pairs including 22 different scenes. In total, 2103 image pairs were used as the training dataset and 1111 pairs of images were used as the test dataset. The Real Blur dataset is a large-scale dataset of blurry images and clear images in the real world, which is composed of two subsets of the same image content. The first subset consists of images obtained directly from the camera, representing the original unprocessed images. The second subset comprises images generated after being processed by the camera's image processor. There are 232 scenarios with low light conditions, including nighttime and indoor settings with weak lighting. These scenarios encompass typical real-world scenes. The dataset contains a total of 4738 pairs of images captured in various scenes. The Real Blur dataset serves as valuable research data for evaluating image deblurring techniques based on deep learning models in real-world settings. For the experiments, 3758 image pairs from this dataset were allocated for training the proposed model, while the remaining 980 pairs were reserved for testing and evaluation.

To strengthen the generalization ability of the model, data enhancement operations were performed on the GoPro and Real Blur training datasets. The operations included random rotation and adding Gaussian noise. Specifically, the data augmentation included random flips in both horizontal (left to right) and vertical (upside down) directions, as well as rotations at angles of 90, 180, and 270 degrees. Gaussian noise was also introduced with an average value of 0 and a variance of 0.0001. As a result, the GoPro training dataset was expanded from 2103 image pairs to 8412 image pairs, while the Real Blur training dataset grew from 3785 image pairs to 15,032 image pairs through these augmentation techniques.

In order to prevent model overfitting, images from the training datasets were randomly cropped to a size of 256 × 256 pixels. The training period was set to 3000 rounds and the initial learning rate was set to 1e-4, which was halved every 1000 rounds. The batch size was also set to 10. The adopted network optimization method was Adam^24^, with the parameters of ${\beta }_{1}$ = 0.9 and ${\beta }_{2}$ = 0.999. To expedite the experimental training, the model was trained using a GPU, which is well-suited for computationally intensive image processing tasks. The experimental environment and configuration, as detailed in Table 1, were employed in the present research.

**Table 1:** Experimental environment and configuration.

Experimental environment	Configuration
Operating system	Windows10
GPU	GTX 2080
Memory size	16.0 GB
Programing language	Python
Deep learning framework	PyTorch

### Design and comparative analysis of loss function

#### Design of loss function

The MSE (mean square error) can allow for the difference between predicted and actual values to be obtained, and is widely used as the loss function for model evaluation. In the present study, MSE was used for model training. The mapping function is shown in Eq. (15).

15 $$D\left(b\right)=\widehat{S}$$

where D indicates the mapping function; b indicates the blurred image; and Ŝ represents an image restored from a model. The MSE loss function is shown in Eq. (16).

16 $${L}_{MSE}\left(\theta \right)=\frac{1}{2N}\sum_{i=1}^{N}{\Vert D\left({b}_{i};\theta \right)-{S}_{i}\Vert }_{F}^{2}$$

where D represents the mapping function obtained by learning the model; S represents the label image; θ represents the learning parameters in the network model; and N represents the N pairs of images inputted into the network by the training dataset.

The latest research has shown that there are other auxiliary losses besides the loss of image content. In image enhancement tasks, a common approach involves minimizing the distance loss between the input and output in the feature space. This technique has been extensively adopted and has proven effective in achieving improved results ^25^. There is still a gap between the real image and the restored image, especially in the frequency domain. As is widely known, since the purpose of image deblurring is to recover the lost high-frequency component information, the difference in the frequency domain space should be reduced. Therefore, a frequency reconstruction loss function (FRLF) was proposed in the present study to address the aforementioned problem. The frequency reconstruction loss can be defined as the L1 distance between the real image and the blurred image in the measured frequency. The mathematical expression is shown in Eq. (17).

17 $$L_{{FRLF}} = \frac{1}{{2N}}\sum\limits_{{i = 1}}^{N} {\left\| {\Gamma \left( {\hat{S}} \right) - \Gamma ({\text{S}})} \right\|_{1} }$$

where Г represents Fast Fourier Transform (FFT), which transforms image features from the spatial domain to the frequency domain; Ŝ represents the potential image restored by the model; S represents the label image; and N represents the sum of the logarithms of the image input into the FFT. Hence, the loss function used for model training in the present study was Ltotal, which is shown in Eq. (18). In the experiments, the parameter λ was set to 0.1.

18 $${L}_{total}={L}_{MSE}+\lambda {L}_{FRLF}$$

#### Comparative analysis of loss function

In order to verify the validity of the proposed FRLF, it was compared with two related loss functions, the SSIM loss function and the perceptual loss function^26^. The functions were applied to the proposed model and tested using the GoPro test data set. All loss functions were loaded using the strategy in Eq. (19), where ${L}_{a}$ represents different loss functions. To balance the output of the loss functions and ${L}_{MSE}$, different parameters were assigned to the $\lambda$ of different loss functions. The performances of the different loss functions in the proposed model are shown in Table 2.

**Table 2:** Performance comparison of loss functions.

Loss functions	λ	PSNR(dB)	SSIM
L_MSE_	–	29.46	0.931
L_MSE_ + L_SSIM_	0.2	30.83	0.935
L_MSE_ + L_Perceptual_	0.5	31.31	0.944
L_MSE_ + L_FRLF_	0.1	31.53	0.948

19 $${L}_{total}={L}_{MSE}+\lambda {L}_{a}$$

Table 2 shows that compared with the MSE loss function only, the introduction of the loss function could improve the performance of the model. The SSIM loss function PSNR increased by 1.37 dB, and the SSIM increased by 0.004. The perceptual loss function PSNR was increased by 1.85 dB, and the SSIM was increased by 0.013. The introduction of FRLF delivered the most significant improvements. It led to a substantial PSNR increase of 2.07 dB and a considerable SSIM increase of 0.017. FRLF is particularly effective as it aids the model in recovering high-frequency image details by reducing frequency gaps. Consequently, the conclusion is that FRLF is a straightforward yet highly effective loss function for improving model performance.

### Evaluating indicator

Using PSNR and SSIM as evaluation indicators, the proposed model was quantitatively analyzed using the GoPro and Real Blur test datasets, and compared with other models such as the CNN^27^, the Multi-scale CNN^28^, spatially variant RNN^29^, the improved U-Net model^19^, the CAR GAN^30^, Attentive deep network^6^, and BDIRNet^33^. Those other models were analyzed using the same test datasets with the proposed models. Table 3 shows the results, indicating that the proposed model performed well in terms of the PSNR and SSIM indicators on GoPro.

**Table 3:** Performance comparison of various methods on GoPro.

Method	CNN ^27^	Multi-scale CNN ^28^	Spatially Variant RNN ^29^	Improved U-Net ^19^	CAR GAN ^30^	Attentive deep network ^6^	BDIRNet ^33^	Ours
PSNR(dB)	25.32	29.21	30.24	30.83	31.11	31.23	31.25	31.53
SSIM	0.785	0.930	0.934	0.938	0.914	0.946	0.945	0.948

To rigorously assess the performance of the proposed model, a series of experiments were conducted on the Real Blur test dataset. The model's results were quantitatively compared with those of other existing models, including CNN^27^, Spatially Variant RNN^29^, CAR GAN^30^, and BDIRNet^33^. The results are shown in Table 4, indicating the proposed model performed better in terms of the PSNR and SSIM indicators on Real Blur.

**Table 4:** Performance comparison of various methods on Real Blur.

Method	CNN ^27^	Spatially variant RNN ^29^	CAR GAN ^30^	BDIRNet ^33^	Ours
PSNR(dB)	26.31	29.56	30.69	29.62	31.32
SSIM	0.840	0.909	0.922	0.86	0.934

### Model parameters and efficiency analysis

To thoroughly analyze the scale and efficiency of the proposed model, a comparison was made between its running time and model parameter size in contrast to other methods when restoring images using the GoPro test dataset. The comparison results are shown in Table 5, where Time represents the run time required for the model, and Size represents the model parameters size. As shown in Table 5, the proposed model took less time and had smaller model parameters than those of the CNN^27^, Multi-scale CNN^28^, SRN-DeblurNet^31^, and BDIRNet^33^, but took longer and had more parameters than the improved U-Net^19^. Such findings could be attributed to the model adding more modules and increasing the calculation amount compared with it. Therefore, the proposed model has certain advantages in parameter scale and efficiency.

**Table 5:** Comparison of model running time and model size.

Method	CNN ^27^	Multi-scale CNN ^28^	SRN-DeblurNet ^31^	Improved U-Net ^19^	Attentive deep network ^6^	BDIRNet ^33^	Ours
Time (s)	1200	3.2	3.32	0.56	0.28	1.4	0.67
Size (MB)	50.10	303.60	41.3	22.6	26.34	25.26	24.4

## Discussion

### Visual analysis

To provide further validation of the proposed model’s deblurring effectiveness, this section presents visual results on both the GoPro test dataset and the Real Blur test dataset. The evaluation was conducted by comparing the deblurred images produced by the model to the original clear images, serving as reference, to assess and analyze the quality of image deblurring achieved by the model.

**Figure 9:**
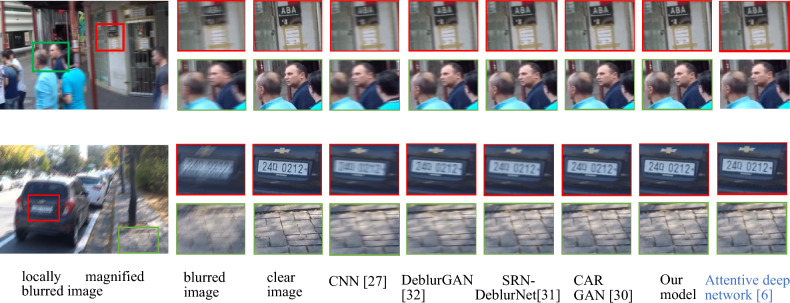
Visual effect comparison on GoPro test dataset.

Figure 9 shows a visual effect comparison on the GoPro test dataset. The images are displayed in a left-to-right sequence, showcasing different aspects of the deblurring process. Starting from the left, there is a magnified view of the initially blurred image. Next, the original clear image serves as a reference for evaluation. Subsequently, the deblurring results from various models are displayed. The restoration image obtained using CNN^27^ is shown, followed by the result from DeblurGAN^32^, SRN-DeblurNet^31^, and CAR GAN^30^. Finally, the far-right image represents the restoration achieved by the proposed model. This visual comparison allows for a direct assessment of the deblurring effectiveness of each method on the GoPro test dataset, providing valuable insights into the quality of image restoration. As shown in Fig. 9, the restoration image generated by the CNN^27^ was juxtaposed with the blurred image. The method exhibited a degree of blurring reduction capability; however, it still manifested noticeable artifacts, resulting in an insufficient deblurring effect. In comparison to CNN^27^, the DeblurGAN^32^ demonstrated an enhancement in deblurring effectiveness but was not entirely free from artifacts. The visual representation of SRN-DeblurNet^31^ showed a lack of conspicuous artifacts, yet it suffered from blurriness in ground-level details. CAR GAN^30^ exhibited superior artifact removal capabilities but falls short in restoring heavily blurred regions. Attentive deep network^6^ have similar deblurring effects to our method, but their size is slightly larger. In contrast, the proposed model excelled in image deblurring, offering the most notable results by capturing extensive image information, achieving clearer image restoration, and displaying reduced susceptibility to artifacts. This capability effectively mitigated interference from artifacts and other factors.

**Figure 10:**
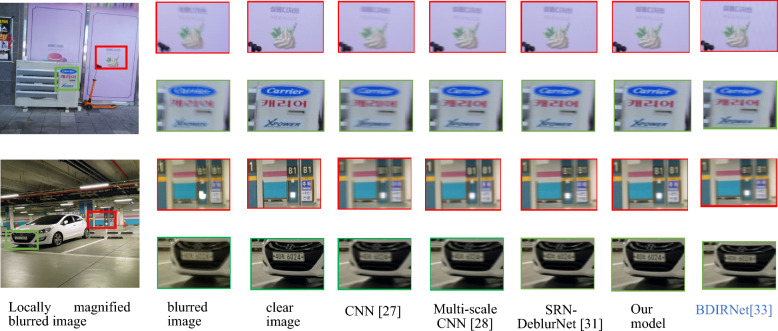
Visual effect comparison on Real Blur test dataset.

Figure 10 illustrates a visual comparison on the Real Blur test dataset. From left to right, the sequence includes a locally magnified blurred image, a clear image, and restoration images generated by CNN^27^, Spatially Variant RNN^28^, SRN-DeblurNet^31^, and the proposed model, respectively.

As depicted in Fig. 10, the restoration image produced by CNN^27^ exhibited some improvement over the blurred image. Nevertheless, it remained considerably blurry, with a noticeable presence of artifacts. Multi-scale CNN^28^ achieved a superior outcome compared to CNN^27^, resulting in a clearer restored image that mitigated the impact of artifacts. However, artifacts were still present in the restored image. SRN-DeblurNet^31^, BDIRNet^33^ demonstrated superior artifact removal capabilities in comparison to Multi-scale CNN^28^, resulting in a clearer restored image. However, the restoration effect for areas with severe blurring was less than ideal. The proposed model performed well on the Real Blur test dataset, achieving effective image restoration with minimal artifacts. Nonetheless, there is still room for improvement when compared to clear images.

### Module performance analysis

To assess the effectiveness of the proposed modules, five model experiments were conducted. Model1 excluded only DWT and IWT. Model2 omitted the DMRFAB module alone. Model3 excluded only the SA and CA modules. In Model4, only the MLFF module was not included. Model5 integrated all proposed modules. The results are summarized in Table 6. In Model2, the image evaluation index PSNR reached 30.07, and SSIM reached 0.928. This suggests that the inclusion of DWT and IWT contributed to the improved performance of Model2. These transformations effectively captured contextual and textural information across different image frequencies. Upon introducing the DMRFAB module in Model3, PSNR increased to 31.28 dB, and SSIM improved to 0.941. This demonstrates the effectiveness of the DMRFAB module in enhancing deblurring capabilities. In Model4, the introduction of the SA and CA modules led to a PSNR of 31.35 dB and SSIM of 0.943. This can be attributed to the synergy between the attention mechanism and convolution, which prioritizes global information and selects crucial feature information. This, in turn, enhanced the model’s deblurring ability. Finally, in Model 5, where all modules were incorporated, PSNR reached 31.53, and SSIM rose to 0.948. This underscores the positive impact of the proposed modules on enhancing the quality of the restored images. The combination of these modules improved feature extraction, facilitated feature reconstruction, and aided the model in learning the mapping relationship between blurry and clear images.

**Table 6:** Quantitative evaluation results of different models.

Method	Model1	Model2	Model3	Model4	Model5(Ours)
PSNR (dB)	28.11	30.07	31.28	31.35	31.53
SSIM	0.891	0.928	0.941	0.943	0.948

## Conclusion

In this paper, an image deblurring method based on U-Net model was proposed, in which an MLFF module and a DMRFAB module was designed. The MLFF module integrates feature information in different layers of the U-Net network, changes the inherent information flow mode in the conventional U-Net network, and integrates feature information of different scales, so that the network can extract more feature information. DMRFAB introduces both a spatial attention mechanism and a channel attention mechanism, explores the relationship between different feature channels and the spatial relationship between different features, overcomes the shortcomings of a single attention mechanism, obtains the information of important parts of the features, and further obtains the deep features, thereby improving the effect of blur removal. Additionally, FFT was introduced into the loss function to obtain the frequency value of the image, reduce the frequency difference of the image, and improve the effect of deblurring. The average PSNR and average SSIM values for the GoPro dataset were 31.53 and 0.948 respectively, while those for the Real Blur dataset were 31.32 and 0.934 respectively, which were higher than those of the other methods. Therefore, the present method can produce a better deblurring effect.

In future work, the focus will center on refining the model. This entails efforts to enhance its lightweight characteristics, thereby optimizing its performance on commonly used mobile devices.

## Data Availability

The datasets generated and/or analyzed during the current study are available from the corresponding author upon reasonable request.
